# Rapid demulsification of pickering emulsions triggered by controllable magnetic field

**DOI:** 10.1038/s41598-020-73551-w

**Published:** 2020-10-06

**Authors:** Hui Yang, Shujuan Wang, Wei Zhang, Jiazhong Wu, Siyu Yang, Danfeng Yu, Xu Wu, Yang Sun, Jinben Wang

**Affiliations:** 1 CAS Key Lab of Colloid, Interface and Chemical Thermodynamics, Institute of Chemistry, Chinese Academy of Sciences, Beijing, 100190 People’s Republic of China; 2 State Key Laboratory of Enhanced Oil Recovery, Research Institute of Petroleum Exploration and Development of PetroChina, Beijing, 100083 People’s Republic of China; 3 Department of Chemistry and Chemical Engineering, Guangzhou University, Guangzhou, 510006 Guangdong People’s Republic of China; 4 Center for Physicochemical Analysis and Measurement, Institute of Chemistry, Chinese Academy of Sciences, Beijing, 100190 People’s Republic of China

**Keywords:** Green chemistry, Physical chemistry

## Abstract

Pickering emulsions with on–off properties provide significant advantages over simple solid-stabilized emulsions for the development of novel materials, such as oil-displacing agents for enhanced oil recovery and templates for the fabrication of porous materials. However, the irreversible adsorption of particles as emulsion stabilizers endows the Pickering emulsions with kinetically stable property, resulting in a huge challenge to break the stability. Here we fabricated microscale Pickering emulsions, by the use of paramagnetic particles, which possess excellent stability for several months and more interestingly perform complete demulsification under controllable magnetic fields in several minutes. The alternating asymmetrical magnetic field endows oil-in-water droplets ‘‘big’’ N and S poles on the outer particle layers, and attracts the solid particles to the bottom of the vial after the coalescence and the deformation of the droplets, bringing the prevention of re-emulsion and the cyclic utilization. This facile strategy to produce stable Pickering emulsions with a magnetic-response opens a promising avenue for various practical applications including oil recovery, wastewater treatment, and sludge removal.

## Introduction

In significant application fields including oil recovery, synthesis of novel materials, and pharmaceutical industry, there is a great need of a facile, efficient, and green strategy to fabricate emulsions with long-term stability and rapid demulsification response on demand^1–5^. Therefore, enormous efforts have been devoted to the development of stimuli responsive emulsions, especially for the ones being responsive to ‘‘physical’’ triggers such as magnetic field^6–9^, temperature^10–12^, or light^13–15^. Without introducing ‘‘chemical’’ demulsifiers to the emulsion systems, the environmental and secondary pollution, as well as the negative impact on downstream production processes, can be completely avoided. However, the research and technology on switchable emulsions are rarely reported^6,8^, except a complete phase separation triggered by the aid of both magnetic field and stirrer, owning to the missing materials and understanding of the responsive demulsification mechanisms.

With this in mind, we imposed a controllable magnetic field on a series of Pickering emulsions stabilized by paramagnetic particles. It was found that the emulsions, showing a long-term stable state, were successfully triggered to a fast and high-efficient demulsification in the presence of an asymmetrical and alternating magnetic field. The effects of the particle concentration and magnetic property, and the magnetic field strength and time-domain characteristic, on the responsive demulsification performance were explored. Our results are expected to provide a scientific basis for a rapid, high-efficient, reproducible, and environmentally friendly separation technology.

## Results and discussion

### Preparation of pickering emulsions based on carbonyl iron particles (CIPs)

**Figure 1:**
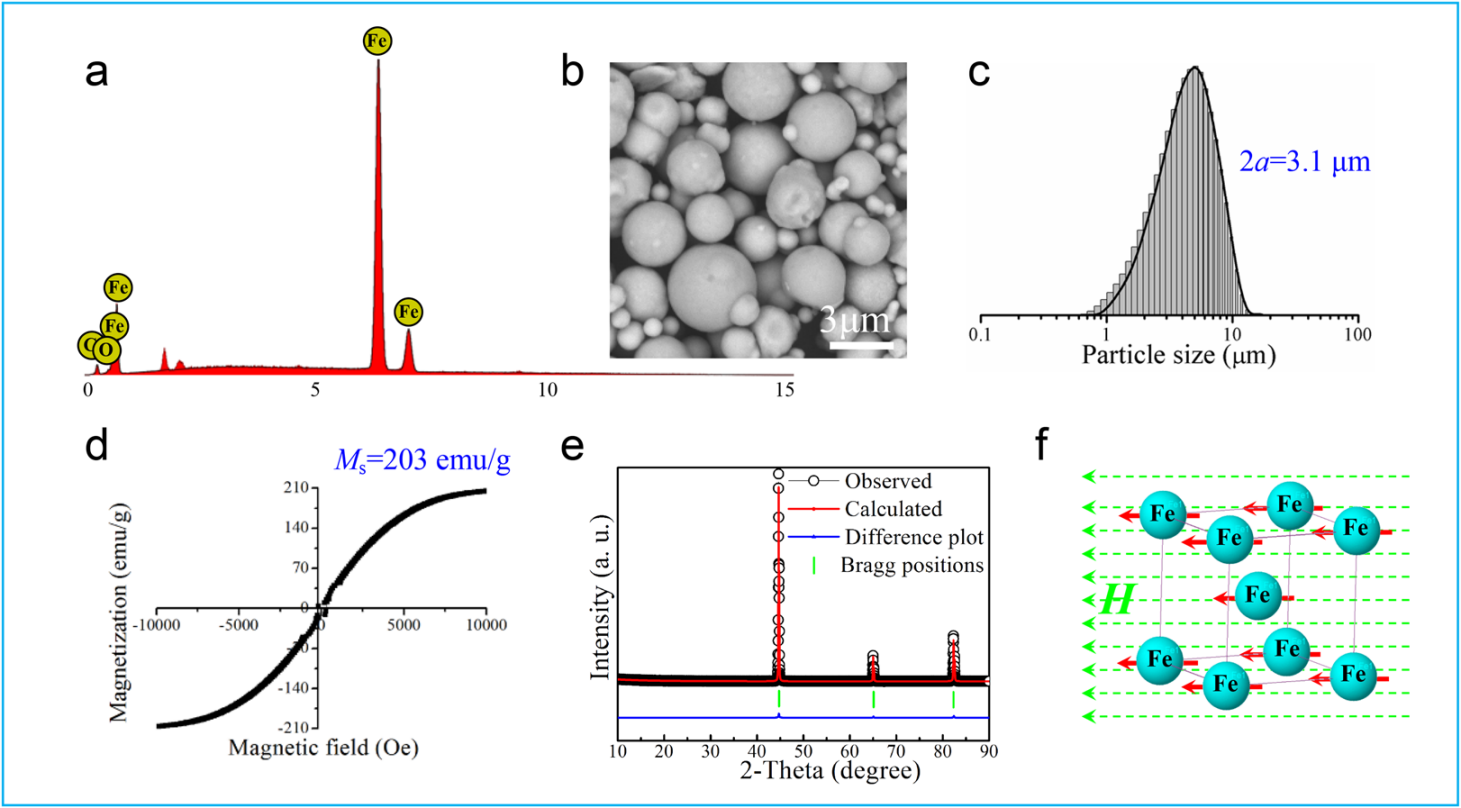
Characterization of carbonyl iron particle (CIP) material: (**a**) EDS spectra, (**b**) SEM image, (**c**) particle size distribution (2*a*: average diameter), (**d**) magnetization curve of CIPs (*M*_s_: saturation magnetization), (**e**) powder XRD pattern of CIP material, and (**f**) magnetic moments of an unit cell in the presence of uniform magnetic field.

CIPs are composed of iron, spherical in shape, and polydisperse in size with an average diameter of 3.1 μm, as shown in Fig. 1a–c. There is no hysteresis or magnetic remanence obtained from the magnetization curve (Fig. 1d), exhibiting a superparamagnetic behavior with a saturation magnetization of 203 emu/g. The characteristic diffraction peaks are located at 44.7°, 65.0°, and 82.3° (Fig. 1e), indexed to the (110), (200), and (211) planes (PDF4 + 2020 No. 00-006-0696) and implied a typical cubic ferrum structure. In the presence of uniform magnetic field, every atom in a single unit cell has the same orientation of magnetic moment and along the direction of the magnetic field (Fig. 1f), being a part of the single magnetic domain.

**Figure 2:**
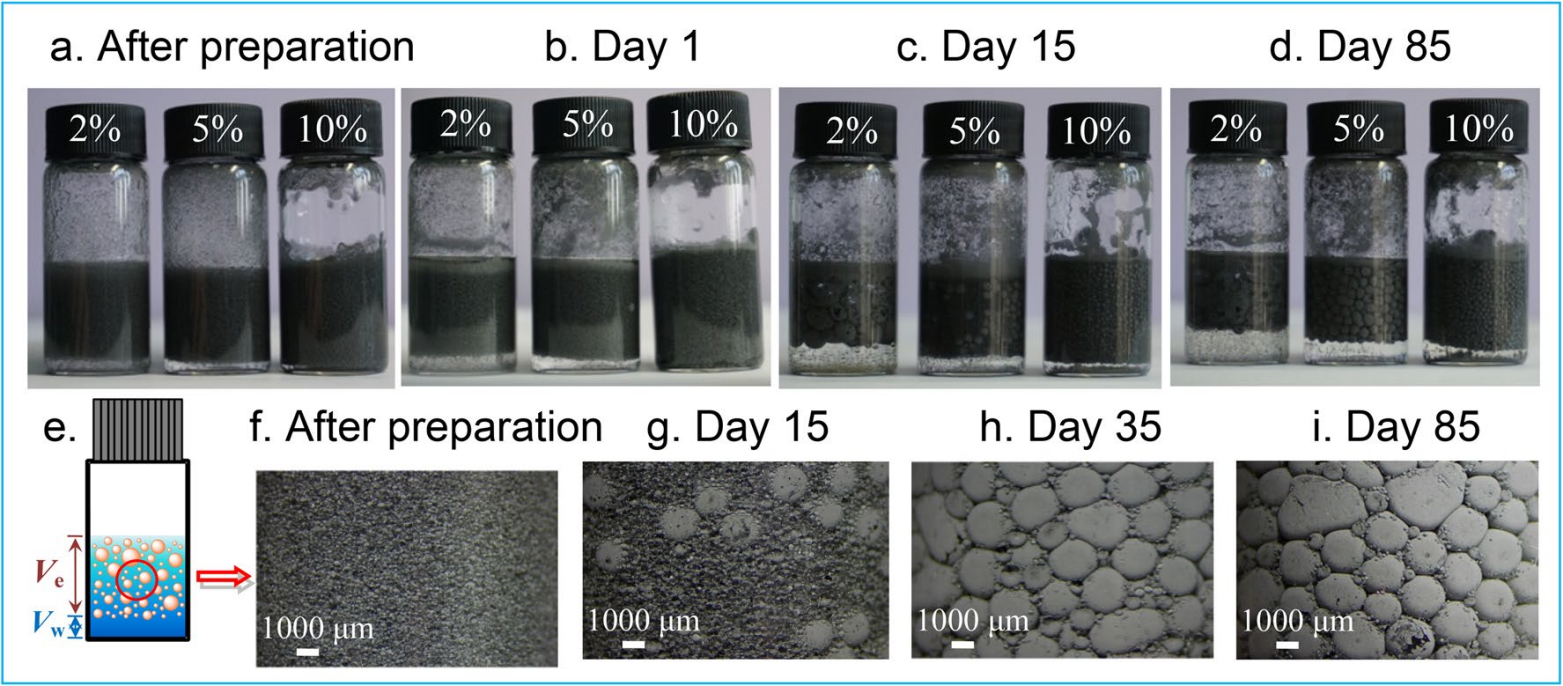
Emulsions stabilized by CIPs after preparation (**a**), and placed for 1 day (**b**), 15 days (**c**), and 85 days (**d**) at different concentrations of 2, 5, and 10 wt%. Stereo microscopic images in situ (**e**) (*V*_e_: emulsion volume and *V*_w_: water volume): (**f**) after preparation, (**g**) placed for 15 days, (**h**) 35 days, and (**i**) 85 days at 5 wt%.

The Pickering emulsions stabilized by 2, 5, and 10 wt% CIPs, respectively, are of O/W emulsion type, showing high conductivity values of the emulsion phase (Table S1) and with a certain distributiuon and composition (Tables S2 and S3). Because the particles can diffuse to the interfacial region, remain there, and form rigid structures, stabilizing the thin films^6,16,17^. The obtained emulsion volume (*V*_e_) increases and the extra water volume (*V*_w_) underneath the emulsion decreases with time or at higher particle concentration (Fig. 2a–e). Compared with the freshly prepared emulsions (Figs. 2f and S1a), the oil droplet size increases largely after 15 days (Figs. 2g and S1b), taking the emulsion with 5 wt% CIPs as an example; interestingly, small oil droplets tend to coalesce to big ones from 15 to 35 days (Figs. 2h and S1c), and the droplet appearance keeps changeless from 35 to 85 days (Figs. 2i and S1d), indicating a good emulsion stability. The formed O/W emulsions are stable attributed to the fact that the hydrophilic particles (Fig. S2) are more easily wetted by the water phase and their contact with the oil phase is reduced, resulting in the curve of interface and the formation of spherical oil droplets^6,8,18^.

### Rapid demulsification triggered by controllable magnetic fields

**Figure 3:**
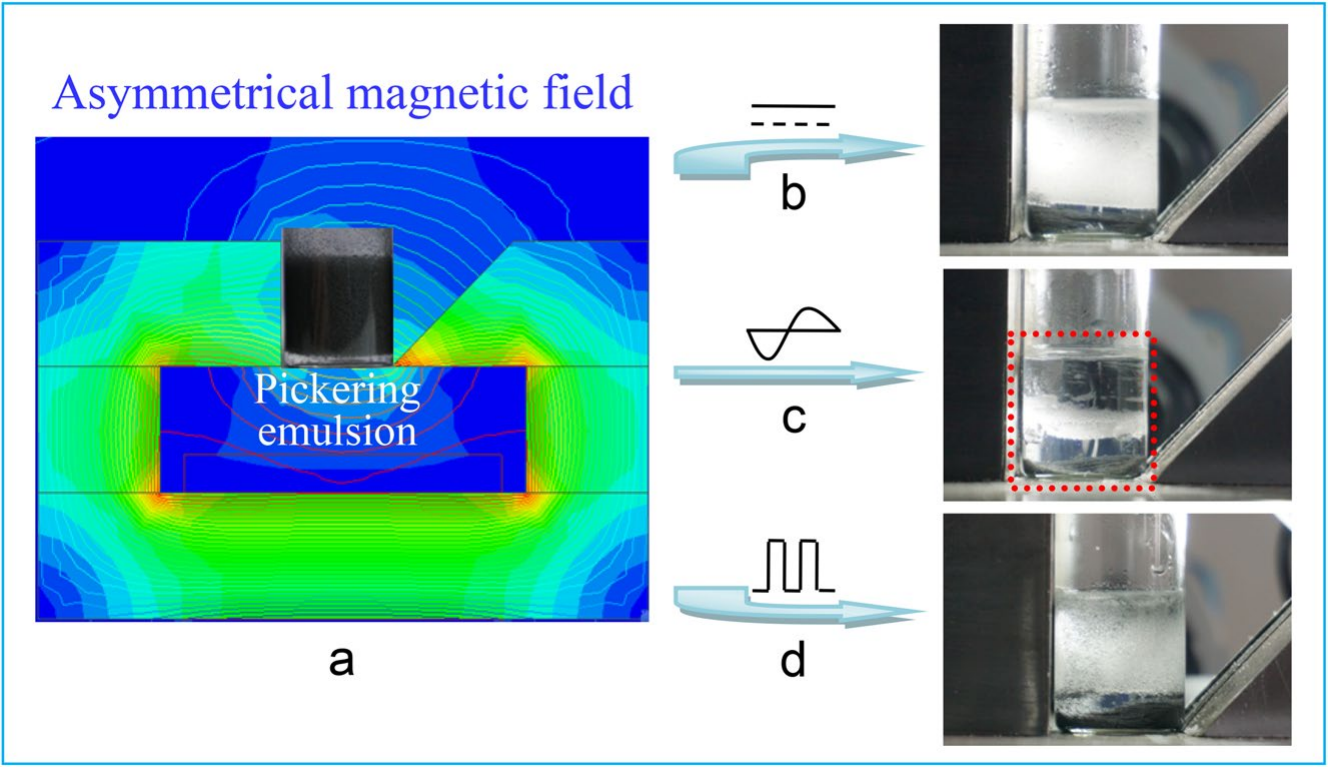
Pickering emulsions stabilized by 5 wt% CIPs under (**a**) steady-state, (**b**) alternating, and (**c**) pulsed asymmetrical magnetic field.

After the introduction of an asymmetrical and non-uniform magnetic field simulated by ANSYS Electronics 18.1 (Fig. 3a), a complete demulsification is triggered within 2–3 min by alternating magnetic field (Fig. 3c and Movie S1), at the particle concentration of 5 wt% and at the exiting current of 12 A. The performance can also be induced by the alternating asymmetrical magnetic field at different particle concentration of 2 and 10 wt% or magnetic field strength at the exiting current of 4, 6, 9 A, as shown in Figs. S3 and S4, respectively. Therefore, the demulsification of the Pickering emulsions in a particle concentration of 2–10 wt% can be triggered in a wide range of magnetic field strength, from 150 to 220 mT (referring to the strongest point of the gradient magnetic field), which is weaker but more efficient than that of NdFeB magnet system in previous report^7,8,19^. Furthermore, based on the designed electromagnetic unit we can describe the magnetic field clearly and discuss the responsive mechanism in detail.

**Figure 4:**
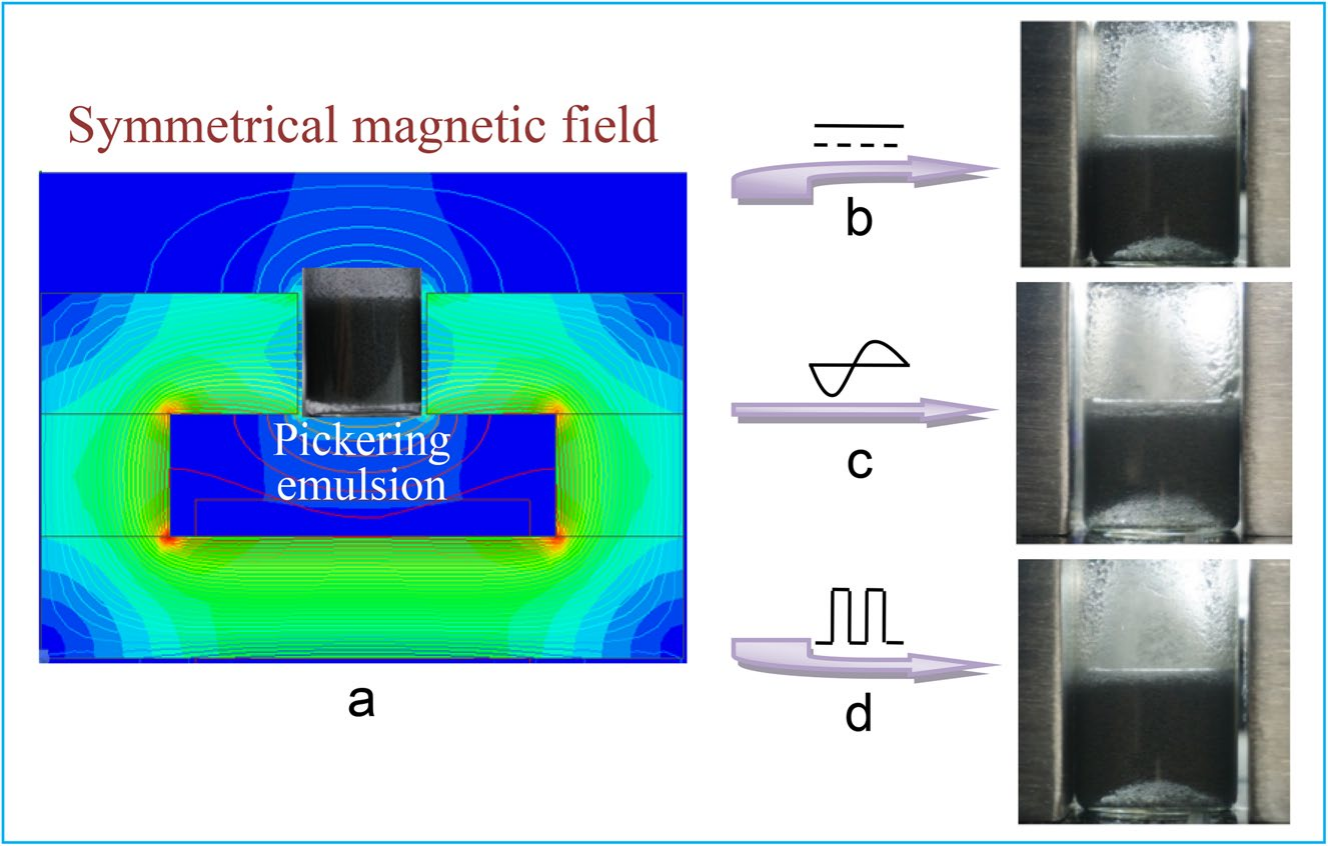
Pickering emulsions stabilized by 5 wt% CIPs under (**a**) steady-state, (**b**) alternating, and (**c**) pulsed symmetrical magnetic field.

In comparison, the complete demulsification is not responsive to a steady-state (Fig. 3b) or pulsed asymmetrical magnetic field (Fig. 3d) and, instead, ‘‘new’’ transparent emulsions are formed with similar drop size (Fig. S5) and stabilized by less CIPs than that of freshly prepared emulsion. In addition, in the presence of a symmetrical and uniform magnetic field (Fig. 4a), a demulsification cannot be triggered by the three kinds of time-domain characteristics (Fig. 4b–d), only resulting in an arched bottom surface and without any change of emulsion within several hours. It suggests that there is an important relationship between particle properties and magnetic field characteristics.

**Figure 5:**
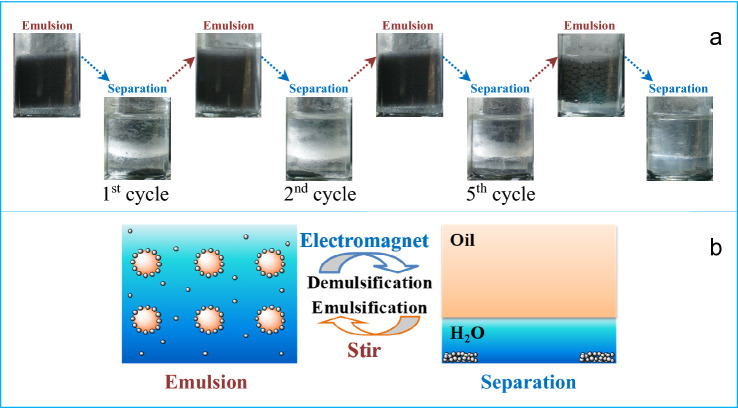
A dozen cycles of emulsification and demulsification: (**a**) experimental photos and (**b**) mechanism sketches.

Interestingly, the recyclability of the Pickering emulsions is performed which is merely responsive to ‘‘physical’’ triggers, taking five cycles as an example as shown in Fig. 5. The complete phase separation occurs using the alternating asymmetrical magnetic field within 10 min, and the destabilized emulsion, with a similar mean drop size (Fig. S6), can be violently re-dispersed using a vortex mixer within 1 min. Compared with conventional chemical emulsifiers, the advantages of our magnetic CIPs are their ability to trigger a complete and rapid phase separation through an external magnetic field, and to be recycled and reused after demulsification. Even stabilized by magnetic nanoparticles (MNPs), Pickering emulsions need both magnetic field and stirrer to induce an oil/water separation^8^, probably because of the 3–4 times lower saturation magnetization of MNPs than that of CIPs.

### Responsive mechanism

**Figure 6:**
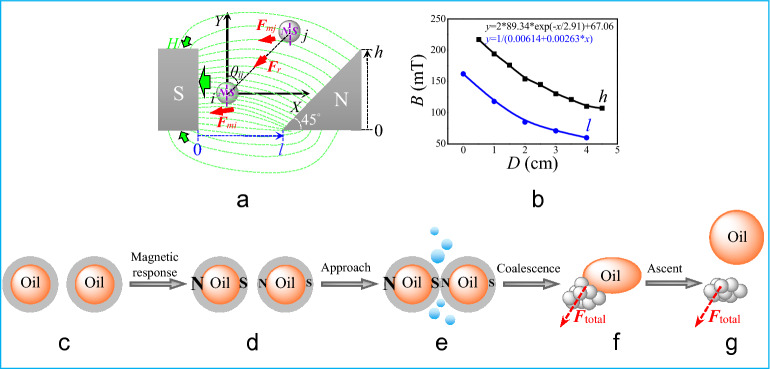
(**a**) Analysis of forces after introducing an asymmetrical electromagnet; (**b**) strength of magnetic field at different width (*l*) and height (*h*) of the triangle magnetic pole; graphical representation of magnetic response mechanism before (**c**) and after (**d**–**g**) introducing the asymmetrical electromagnet.

In the system of Pickering emulsions, the magnetic CIPs situate at oil/water interface and the forces are balanced, including gravity (***F***_g_), buoyancy (***F***_b_), and interfacial tension (***F***_γ_). After the introduction of the electromagnet, the equilibrium state is disturbed, because the external magnetic force (***F***_m_) and interaction force between particles (***F***_r_; see Fig. 6a as an example) are added to the total force (***F***_total_), as follows as Eqs. (1)–(3)^20,21^:

1 $$ {\varvec{F}}_{{{\text{total}}}} = {\varvec{F}}_{{\text{m}}} + {\varvec{F}}_{{\text{r}}} + {\varvec{F}}_{{\text{g}}} + {\varvec{F}}_{{\text{b}}} + {\varvec{F}}_{\gamma } $$

2 $$ F_{{\text{m}}} = mH_{{\text{N}}} - mH_{{\text{S}}} = B_{{\text{N}}} SH_{{\text{N}}} - B_{{\text{S}}} SH_{{\text{S}}} $$

3 $$ F_{{\text{r}}} = \frac{{m_{i} m_{j} }}{{4{\uppi }\mu_{0} r^{2} }} = \frac{{B_{i} B_{j} S^{2} }}{{4{\uppi }\mu_{{0}} r^{2} }} = \frac{{\mu_{{0}} (H_{i} + M_{i} )(H_{j} + M_{j} )S^{2} }}{{4{\uppi }r^{2} }} $$

where *m* represents the magnetic flux and equals to *BS*; *B* represents the intensity of magnetic induction and equals to *μ*_0_(*H* + *M*), which can be measured by a teslameter; *S* represents the superficial area of particle, which can be calculated based on the radius of the particle; *H* represents the strength of magnetic field and equals to *B*/*μ*_a_; *μ*_a_ represents the absolute magnetic conductivity; *M* represents the intensity of magnetization, as shown in Fig. 1d.

**Figure 7:**
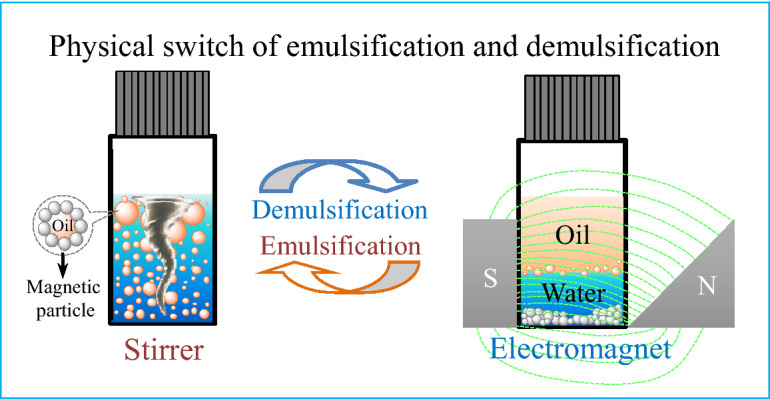
The graphic of responsive destability mechanism in the case of a designed magnetic field.

In the presence of magnetic field, each CIP can be magnetized to a magnetic pole possessing an N and S pole along the magnetic lines, covering a model droplet interface as shown in Fig. S7. In a single particle, the external magnetic force (*F*_m_) can be considered to be zero, according to the little difference in pairs of forces and the little difference in *B* and *H* values in a tiny magnetic pole (Eq. 2), in a uniform magnetic field (Fig. S4a). Meanwhile, every couple of the interaction forces such as *F*_r1_ and *F*_r2_ on the inner layer CIPs can balance each other and, therefore, the droplet as a whole is proposed to own ‘‘big’’ N and S poles on the outer layers. There are two main interaction forces including *F*′_r1_ and *F*′_r2_ imposed on the middle droplet and balanced out because of the uniform magnetic field, which supports the experimental results that there is no responsive phase separation to such kind of magnetic field (Fig. 4). In the case of a non-uniform magnetic field, the magnetic induction intensity (*B*) decreases with the height (*h*) of magnetic pole and the width (*l*) of air gap, as shown in Fig. 6b. Therefore, the interaction force between CIPs *F*_r5_ is stronger than that of *F*_r6_ and the interaction force between droplets *F*′_r3_ is stronger than that of *F*′_r4_ (Fig. S7b), resulting in the magnetic attractions increase along the gradient-enhanced direction of the vial. Droplets attract with each other, merge into a big one, and finally float up to the bulk surface. Compared with no introduction of the magnetic field (Fig. 6c), under the magnetic field and during such process, CIPs aggregate to one side of the droplet and submerge to the bottom of the vial, resulting in the stretching and floating of oil droplets (Figs. 6d–g and S8). The alternating asymmetrical magnetic field can bring the CIPs an initial velocity to overcome the water resistance and the viscoelasticity of the interfacial film. Furthermore, the attraction of CIPs at the bottom prevents oil and water phases from re-emulsion and contributes to the rapid demulsification (Fig. 7). In comparison, in the presence of pulsed asymmetrical magnetic field, the magnetic forces on CIP periodically appear with the presence of magnetic field, resulting in the disturbance of the balance of ***F***_g_, ***F***_b_, and ***F***_γ_ in Pickering emulsion systems. Some of the CIPs aggregate and submerge to the bottom of the vial (Fig. 3d), leaving transparent emulsion with a similar drop size distribution with that before the introduction of magnetic field (Fig. S5). The result is different from previous report, such as the case of the electromagnetic field as a function of the distance to the bottom of the vial^6^. Our work contributes to understanding the responsive destability mechanism in the case of a designed magnetic field for the first time, and provides an effective method for controlling the emulsion stability through particle concentration and saturation magnetization as well as the magnetic field characteristics.

## Conclusions

Our results suggest that the Pickering emulsions stabilized by CIPs exhibit an outstanding stability and a rapid three-phase separation response to a designed magnetic field. The magnetic-response behavior of Pickering emulsion systems can be shown in a broad range of particle concentrations and by the use of paramagnetic CIPs with strong saturation magnetization. By introducing the alternating uniform magnetic field, oil-in-water droplets are endowed with ‘‘new’’ N and S poles and merged into big droplets, leading to the separation of oil and water and the attraction of CIPs to the bottom. By using a stirrer, the components are re-dispersed and the emulsion is re-produced, which in turn can be re-triggered of the responsive demulsification without long-lasting effects. The facile way of controlling the stability of Pickering emulsions in the presence of controllable magnetic field is expected to be a new idea for the development of regulatory strategy and mechanism.

## Materials and methods

CIPs were acquired from Jiangsu Tianyi Ultra-fine Metal Powder Co., Ltd. The surficial morphologies of CIPs samples were observed by using a Phenom ProX scanning electron microscope (SEM, Phenomworld, Netherlands) and an energy dispersive spectrometer (EDS) was equipped for elemental analysis in a surface scanning model^22^. A thin layer of gold was sprayed on the surface prior to SEM observation^22^. Size distribution of CIPs was measured using GSL-101BI Laser particle size meter (Liaoning instrument Research Institute Co., Ltd., China) at room temperature. The magnetic property was measured with a PPMS-9 magnetometer/susceptometer (Quantum Design Inc., America). The measurements were conducted at room temperature with a magnetization field strength cycling from − 10,000 to 10,000 Oe and back to − 10,000 Oe. Water contact angle (CA) of surfaces pressed by CIPs was measured by the CA goniometer (Attension Theta, Biolin Scientific, Sweden) with a sessile-drop method. During the experiment, 2 μL water drop gradually approached and remained on the surfaces with an aging time of about 5 min^21^. The CA values were obtained from video snapshots using a tangent-fitting method in data analysis software (OneAttension, Version 3.2, https://biolinchina.com/product/theta-lite#section6 (URL))^23^. The X-ray diffraction (XRD) pattern of CIPs was recorded by the D/max 2500 diffractometer (Rigaku, Japan) with Cu Kα radiation (*λ* = 0.1541 nm) at room temperature. The measurements were performed at 40 kV and 200 mA from 10° to 90° with a 2*θ* scanning rate of 1°/min^22^. The resultant spectral pattern was compared with the standard pattern of ferrum as a reference^24^.

Emulsions were prepared using the mixture of n-dodecane (J&K Scientific Ltd., AR, 98%) and water in a 2:1 volume ratio, and stabilized by CIPs at the particle concentration of 2–10 wt% which was initially dispersed in n-dodecane. The mixture was vibrated using a DMT-2500 multitube vortex mixer (Hangzhou Mio Instrument Co., Ltd., China) at a speed of 2500 rpm for about 1 min. Millipore Milli-Q grade water (18.2 MΩ cm) was used in all our experiments and all the measurements were carried out under room temperature.

Magnetic fields are produced by a rectangular magnetic loop magnetic field generator with a square side length of 50 mm and a controllable air gap of 5–60 mm (Fig. S9). The shape of magnetic pole beside the air gap can be changed to be rectangle or triangle, in order to produce magnetic fields with controllable strength and distribution. The magnetic field is energized by three groups of coils, each of which consist of 700 turns of 1 mm^2^ copper wire and wraps the copper tube for circulating cooling water. The exciting current of the driving coil can be adjusted in the range of 0–12 A and can be turned to steady-state or pulsed wave, and rectangular or sine wave and, therefore, corresponding magnetic fields are generated.

## Supplementary information

Supplementary Information.

Supplementary Video.
