# Supplementary material for: Rapid demulsification of pickering emulsions triggered by controllable magnetic field
Source: Sci Rep. 2020 Oct 6;10:16565. doi: 10.1038/s41598-020-73551-w (PMC7538568; doi:10.1038/s41598-020-73551-w)
Supplement: Supplementary file 1 — Supplementary Information. [file 41598_2020_73551_MOESM1_ESM.docx]

Supporting Information

Rapid Demulsification of Pickering Emulsions Triggered by Controllable Magnetic Field

*Hui Yang,^*,#,a^ Shujuan Wang,^#,a^ Wei Zhang,^a^ Jiazhong Wu,^b^ Siyu Yang,^b^ Danfeng Yu,^c^ Xu Wu,^c^ Yang Sun,^d^ Jinben Wang^a^*

^a^CAS Key Lab of Colloid, Interface and Chemical Thermodynamics, Institute of Chemistry, Chinese Academy of Sciences, Beijing 100190, P. R. China

^b^State Key Laboratory of Enhanced Oil Recovery, Research Institute of Petroleum Exploration and Development of PetroChina, Beijing 100083, P. R. China

^c^Department of Chemistry and Chemical Engineering, Guangzhou University, Guangzhou, Guangdong 510006, P. R. China

^d^Center for Physicochemical Analysis and Measurement, Institute of Chemistry, Chinese Academy of Sciences, Beijing 100190, P. R. China

Table S1 Conductivity values in the presence of CIPs at different concentrations.

| Particle Concentration wt% | Conductivity  μS/cm |
| --- | --- |
| 2 | 0.232 |
| 5 | 0.835 |
| 10 | 0.340 |

Table S2 Distribution of Particles in the Dodecane-in-Water Pickering Emulsions Stabilized by CIPs Initially in Oil.

| Particle concentration/wt% | m_po_/g | m_pa_/g | m_pe_/g | X/% |
| --- | --- | --- | --- | --- |
| 2 | 0.061 | 0.007 | 0.054 | 88.5 |
| 5 | 0.158 | 0.022 | 0.136 | 86.1 |
| 10 | 0.333 | 0.083 | 0.25 | 75.1 |

Table S3 Composition of the Dodecane-in-Water Pickering Emulsions Stabilized by CIPs Initially in Oil.

| Particle concentration/wt% | V_e_/mL | φ_we_/vol % | φ_oe_/vol % | m_pe_/g | φ_p_/g  (100 mL^-1^) |
| --- | --- | --- | --- | --- | --- |
| 2 | 5.4 | 26.0 | 74.0 | 0.054 | 1 |
| 5 | 5.7 | 29.8 | 70.2 | 0.136 | 2.4 |
| 10 | 6 | 33.3 | 66.7 | 0.25 | 4.2 |

(a) (b)

(c) (d)

Figure S1. Drop size distributions of emulsions stabilized by CIPs after preparation (a), and placed for 15 day (b), 35 days (c), and 85 days (d) at 5wt%.


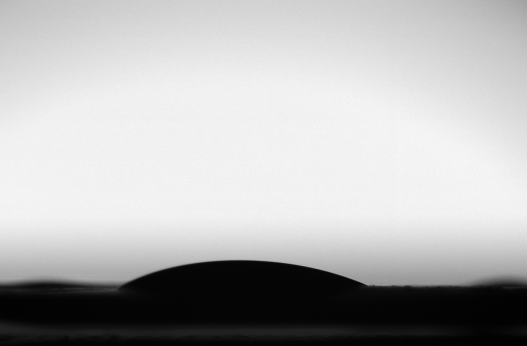


24°

Figure S2 Contact angle (CA) of surfaces pressed by CIPs.


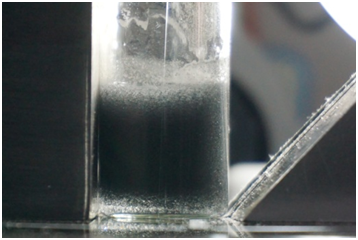

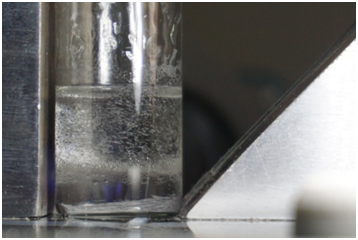


Pickering emulsions

After demulsification

**2%**

**2%**

5 min

6 min


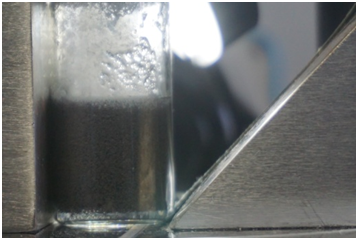

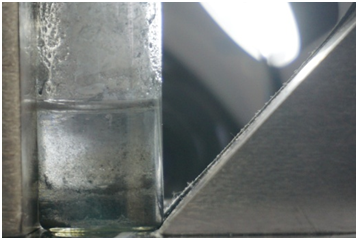


**10%**

**10%**

**a**

**b**

Figure S3 Pickering emulsions stabilized by CIPs under an alternating asymmetrical magnetic field at different concentrations of (a) 2 wt% and (b) 10 wt%.


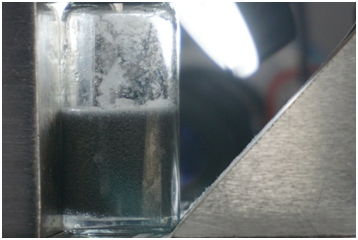

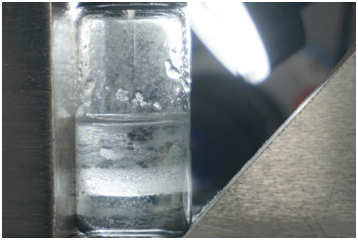

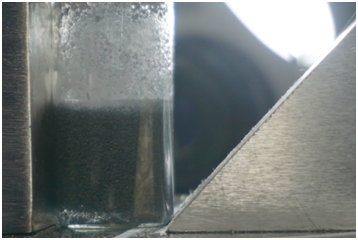

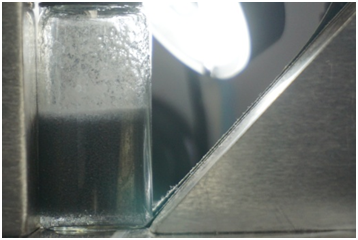

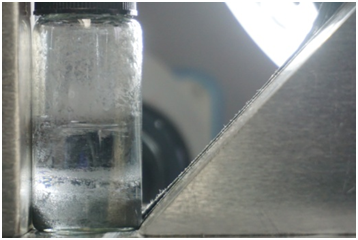


6 min

14 min

22 min

9A 0.2Hz

6A 0.2Hz

4A 0.2Hz

Pickering emulsions

After demulsification


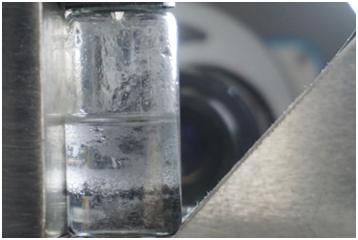


**a**

**b**

**c**

Figure S4 Pickering emulsions stabilized by 5 wt% CIPs under an alternating asymmetrical magnetic field at different currents of (a) 9A, (b) 6A, and (c) 4A.

(a) (b)

Figure S5. Drop size distributions of emulsions stabilized by CIPs after preparation (a), and under pulsed asymmetrical magnetic field (b).

Figure S6. Mean drop size after each cycle of destabilization and reformation.

Oil droplet

CIPs

***F***_r1_

***F***_r2_

***F***_r3_

***F***_r4_

***N***

***S***

***F****'*_r1_

**N S**

**N S**

**N S**

**N S**

**N S**

**N S**

**N S**

**N S**

**N S**

**N S**

**N S**

**N S**

**N S**

**N S**

**N S**

**N S**

**N S**

**N S**

**N S**

**N S**

**N S**

**N S**

**N S**

**N S**

**N S**

**N S**

**N S**

**N S**

**N S**

**N S**

**N S**

**N S**

**N S**

**N S**

**N S**

**N S**

**N S**

**N S**

**N S**

**N S**

**N S**

**N S**

**N S**

**N S**

**N S**

**N S**

**N S**

**N S**

**N S**

**N S**

**N S**

**N S**

**N S**

**N S**

**N S**

**N S**

**N S**

**N S**

**N S**

**N S**

**N S**

**N S**

**N S**

**N S**

**N S**

**N S**

**N S**

**N S**

**N S**

**N S**

**N S**

**N S**

**N S**

**N S**

**N S**

***F****'*_r2_

a. Uniform magnetic field

***F***_r5_

***F***_r6_

***F***_r7_

***F***_r8_

***N***

***S***

***F****'*_r3_

**N S**

**N S**

**N S**

**N S**

**N S**

**N S**

**N S**

**N S**

**N S**

**N S**

**N S**

**N S**

**N S**

**N S**

**N S**

**N S**

**N S**

**N S**

**N S**

**N S**

**N S**

**N S**

**N S**

**N S**

**N S**

**N S**

**N S**

**N S**

**N S**

**N S**

**N S**

**N S**

**N S**

**N S**

**N S**

**N S**

**N S**

**N S**

**N S**

**N S**

**N S**

**N S**

**N S**

**N S**

**N S**

**N S**

**N S**

**N S**

**N S**

**N S**

**N S**

**N S**

**N S**

**N S**

**N S**

**N S**

**N S**

**N S**

**N S**

**N S**

**N S**

**N S**

**N S**

**N S**

**N S**

**N S**

**N S**

**N S**

**N S**

**N S**

**N S**

**N S**

**N S**

**N S**

**N S**

***F****'*_r4_

b. Non-uniform magnetic field

***F***_r1_ = ***F***_r2_ = ***F***_r3_ = ***F***_r4_; ***F****'*_r1_ = ***F****'*_r2_

***F***_r5_ > ***F***_r6_ > ***F***_r7_ > ***F***_r8_; ***F****'*_r3_ > ***F****'*_r4_

Figure S7 Corresponding positioning of particles at a model droplet interface giving a solid-stabilized oil-in-water emulsion and analysis of forces after introducing (a) an uniform magnetic field and (b) a non-uniform magnetic field.

| a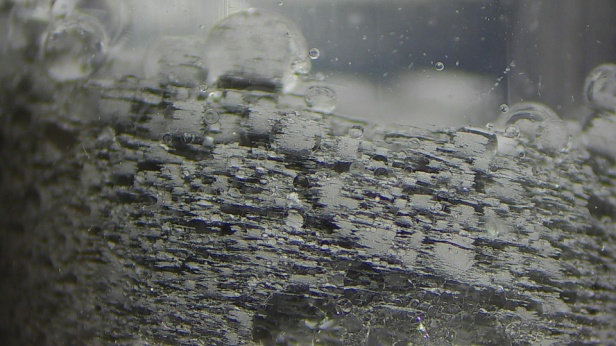 | b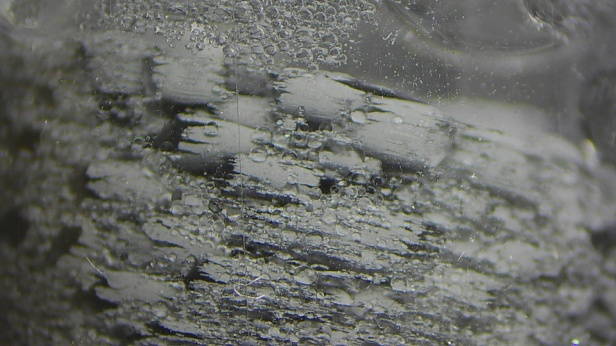 | c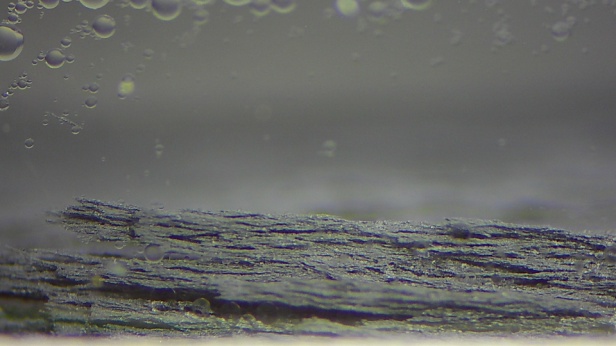 |
| --- | --- | --- |
| Figure S8 Bottom vial area under magnetic field over time. | | |

Figure S9 Setup scheme of the electromagnet field generator.
